# Talent cultivation in health technology assessment: an expert survey

**DOI:** 10.1186/s12909-022-03214-z

**Published:** 2022-03-08

**Authors:** Yu-Shan Wu, Chiehfeng Chen, Lin-Chien Wang, Li-Shan Jian, Yu Ko

**Affiliations:** 1 Department of Pharmacy, College of Pharmacy, Taipei Medical University, Taipei, Taiwan; 2 Division of Plastic Surgery, Department of Surgery, Evidence-Based Medicine Center, Wan Fang Hospital, Taipei Medical University, Taipei, Taiwan; 3 Department of Public Health, School of Medicine, College of Medicine, Cochrane Taiwan, Taipei Medical University, Taipei, Taiwan; 4 Division of Health Technology Assessment, Center for Drug Evaluation, Taipei, Taiwan; 5 Research Center for Pharmacoeconomics, College of Pharmacy, Taipei Medical University, Taipei, Taiwan

**Keywords:** Health technology assessment, Expert survey, Questionnaire

## Abstract

**Background:**

Health technology assessment (HTA) has become essential in many countries over the past few years, and the demand for HTA professionals has increased in academia, governments, and industries. This study aimed to examine which courses are most important and which training activities are most helpful for the development of HTA proficiency as perceived by HTA experts.

**Methods:**

The survey questionnaire was developed by literature review and expert opinion. Convenience sampling was used to survey HTA experts from the industrial sector, academic/research units, and government/independent assessment organizations using an online survey tool, SurveyCake. We collected respondents’ demographic information and asked them to assess the importance of each course included in an HTA program on a 5-point Likert Scale (1 = least important; 5 = highly important). In addition, respondents were asked to assess the extent to which various activities are helpful for HTA proficiency development.

**Results:**

A total of 158 domestic and overseas experts in HTA-related fields were invited to participate in the survey and 68 completed the questionnaire. Among the respondents, the majority were female (57.4%) and working in academia (44.1%). The mean ± standard deviation of respondents’ age and number of years spent in HTA-related fields were 43.2 ± 11.0 years and 11.3 ± 9.9 years, respectively. The course that was rated the most important was “Pharmacoeconomics/Cost-effectiveness analysis” with a score of 4.8 ± 0.4 points, followed by “Health economics” at 4.7 ± 0.7 points. Moreover, internships at HTA-related institutions were perceived to be the most helpful training activity for HTA proficiency development.

**Conclusions:**

Our study findings provide a better understanding of the requirements for developing HTA proficiency and can serve as a reference for the modification of current HTA education and training programs.

**Supplementary Information:**

The online version contains supplementary material available at 10.1186/s12909-022-03214-z.

## Background

Health technology assessment (HTA) is a multidisciplinary process that uses explicit methods to determine the value of a health technology at different points in its lifecycle. The purpose is to inform decision-making in order to promote an equitable, efficient, and high-quality health system [1]. There are two major domains of HTA: (1) clinical evaluation such as efficacy analysis, and (2) economic evaluation such as cost-effectiveness analysis and budget impact analysis. The primary objective of HTA is to inform policy decision making [2]. Policymakers can use HTA results to better understand the value of a health technology, deal with its uncertainties, manage its opportunities and challenges, and make appropriate resource allocation. In Taiwan, the main HTA executor is the HTA Division within the Center for Drug Evaluation (CDE), Taiwan [3], which was established on Apr 1, 2008, and is funded by the Ministry of Health and Welfare. The HTA Division’s mission is to establish a transparent, evidence-based health technology assessment system in Taiwan, and its HTA analysis results have been used to assist decision-making related to new drugs’ reimbursement coverage by the National Health Insurance [4].

Despite HTA’s increasing global importance over the years [5, 6], no school had yet established a graduate or credit-certificate program to cultivate talent for domestic HTA needs in Taiwan until 2017. To respond to the increasing demand for HTA proficiency and with the sponsorship of the CDE, Taiwan, the Health Technology Assessment Credit-Certificate Program (hereinafter referred to as “the HTA program”) was first established at Taipei Medical University (TMU) in 2017. The program integrated university HTA-related courses to cultivate HTA proficiency and promote HTA-related research [7]. All registered TMU undergraduate and graduate students were eligible to apply for the HTA program, and the applicants could obtain a certificate upon successful completion of 16 required credit units, or about 8 courses. The required credits include 4 credits for the Basic Module, 6 credits for the Core Module, and 6 credits for the Application Module. The Basic Module consists of two mandatory courses: biostatistics and epidemiology. The Core Module has three subject areas, and students need to obtain two credits in each area: (1) health economics / pharmacoeconomics, (2) evidence-based medicine, and (3) pharmacy administration / health care policy. In the Application Module, students were able to choose from a list of more than ten courses.

Currently, HTA is still in its developing stage in Taiwan; hence, there remain difficulties, such as implementation, recruitment, and talent cultivation, in different HTA fields. This study aimed to provide a better understanding of the problems and challenges faced by academia, industry, and government. Moreover, considering the increasing demands for HTA proficiency, by collecting HTA experts’ input, we aimed to assess whether the courses provided in our HTA program are truly useful and what kinds of activities are helpful for HTA proficiency development. The findings will serve as a good reference for HTA education and training programs.

## Methods

This was a cross-sectional survey conducted between January and March 2019 that used a structured online questionnaire to investigate the opinions of HTA professionals in academia, industry, and government (including independent assessment organizations). This study was approved by the Taipei Medical University-Joint Institutional Review Board (Approval number: N201812049).

### Survey questionnaire

A questionnaire designed specifically for this study was developed by literature review and expert opinion. The questionnaire was drafted by study investigators and reviewed by nine HTA experts working in academia, in the industrial sector, or for the government. These experts included professors with experience in pharmacy practice and/or HTA research, researchers with expertise in questionnaire development and survey research, senior researchers from the CDE’s HTA Division, and a market access manager from a pharmaceutical company. These expert reviewers were asked to improve the clarity, relevance, and organization of the survey questionnaire. Several rounds of revisions were done after receiving the experts’ comments on the content, wording, and layout of the questionnaire. Specifically, the definition of HTA was updated, several similar courses were combined into one rating item to avoid redundancy and response fatigue, the layout of the questionnaire was revised for easier reading and answering, and several ambiguous words and phrases were clarified. The final version was available in both Chinese and English, and it consisted of three sections: (1) personal information, (2) evaluation of the importance of HTA-related courses and the helpfulness of certain activities for HTA proficiency development, and (3) difficulties encountered in conducting HTA. In the questionnaire, respondents were asked to specify their work setting: academic (including research units), industrial (i.e., pharmaceutical and consulting companies), or governmental (including independent assessment organizations). For the evaluation of importance/helpfulness, respondents were asked to rate each course/activity on a 5-point Likert scale, where 5 = highly important/very helpful and 1 = least important/not at all helpful. Moreover, respondents were asked to select the top 5 courses that were most helpful to HTA personnel in their field. The top 1^st^, 2^nd^, 3^rd^, 4^th^, and 5^th^ courses chosen were given a score of 5, 4, 3, 2, or 1 point(s), respectively, and a total score was calculated for each evaluated course, where the higher the score, the more helpful the course.

### Data collection

The online version of the questionnaire was developed using SurveyCake, a premium online survey tool. Convenience sampling was used to survey domestic and overseas HTA experts from the industry, academic/research units, and government/independent assessment organizations. The names and emails of experts surveyed were obtained from the websites of HTA-related associations and conferences as well as the investigators’ personal contacts. An invitation email with a survey link was sent to the experts in January 2019. To encourage participation, a reminder email was sent out twice after the first email.

### Statistical analysis

Survey responses were analyzed using SPSS 19.0. Descriptive statistics were used to summarize respondents’ demographics. The ratings for the importance of each course and the helpfulness of each activity are presented by means and standard deviations. Frequencies and percentages were used to reveal the difficulties encountered by respondents in their own fields. In addition, the Kruskal–Wallis test was performed to examine differences in the importance of the courses among experts in different fields. Statistical significance was set at *P* < 0.05.

## Results

### Respondent demographics

One hundred and fifty-eight domestic and overseas HTA experts with a valid email address were selected for participation, and 68 (43.0%) completed the survey. The characteristics of respondents are summarized in Table 1. Among the respondents, 57.4% were female, 58.8% were from Taiwan, 54.4% had a PhD degree, and 44.1% were working in academia. In addition, the respondents’ mean age was 43.2 years (SD = 11.0), and they had worked in HTA-related fields for a mean of 11.3 years (SD = 9.9).

**Table 1:** Respondent characteristics

Variables	Mean(SD)
Age (years)	43.2 (11.0)
Working in HTA-related fields (years)	11.3 (9.9)
**Variables**	No. (%)
Gender	
Female	39 (57.4%)
Male	13 (42.6%)
Education	
College	3 (4.4%)
Masters and equivalent	28 (41.2%)
PhD and equivalent	37 (54.4%)
Specialized Fields	
Industry	15 (22.1%)
Government	23 (33.8%)
Academia	30 (44.1%)
Country	
Taiwan	40 (58.8%)
Others	28 (41.2%)

### HTA course assessment

Table 2 presents the assessment results of the importance of each course offered in the HTA program. Overall, the course that was rated by all respondents as the most important was Pharmacoeconomics (mean ± SD = 4.80 ± 0.41), followed by Health Economics (4.68 ± 0.68) and Epidemiology (4.50 ± 0.74). When the respondents were asked to select the top five courses that were most helpful to HTA personnel in their field, the total score showed that Pharmacoeconomics, Health Economics, and Epidemiology were ranked among the top four courses by those working in academia, industry, and government. Furthermore, Biostatistics and Evidence-based Medicine were ranked 3^rd^ by academics and government workers, respectively, and Economic Evaluation of Health Care Policy was ranked 2^nd^ by those working in the industrial sector.

**Table 2:** The assessments of the importance of HTA courses

**Courses**	**Mean**	**SD**	**Academia** (*n* = 30)	**Government** (*n* = 23)	**Industry** (*n* = 15)
Pharmacoeconomics & outcomes research / Cost-effectiveness analysis / Pharmacy administration & pharmacoeconomics	4.80	0.41	84	64	46
Health economics	4.68	0.68	71	39	31
Epidemiology	4.50	0.74	50	45	30
Health outcome research	4.47	1.00	37	18	19
Biostatistics	4.44	0.67	56	33	6
Evidence-based medicine / Application of clinical evidence-based medicine	4.41	0.70	42	42	13
Economic evaluation of health care policy	4.34	0.70	32	35	44
Clinical evidence-based drug evaluation	4.26	0.80	7	23	9
Patient-reported outcomes measurement / Health indicators & measurement	4.21	0.87	16	4	7
Clinical trials	4.00	0.69	12	15	3
Academic writing	3.71	0.90	6	8	2
Drug information	3.69	0.87	7	2	3
Application of statistical software	3.63	0.96	8	3	0
Pharmacy administration & law / Regulations of medicinal product registration	3.43	0.90	4	4	3
Concept of management	3.00	0.99	2	0	4
Public relations & mass communication	2.91	0.99	0	1	5

### Helpfulness of training activities

The assessment of the helpfulness of several types of training activities revealed that internships at institutions related to HTA are the most helpful (mean ± SD = 4.26 ± 0.80), followed by inviting domestic and overseas professionals to offer short-term training courses on HTA-related topics (4.18 ± 0.90). These two activities consistently had the highest rating scores among respondents from all three fields. The helpfulness of other training activities such as HTA student clubs/associations and visiting HTA-related agencies received lower ratings.

### Difficulties in HTA

As shown in Table 3, more than half of the respondents (*n* = 36, 52.9%) considered “Lack of local data” to be a difficulty they have encountered. Other difficulties reported by more than two-fifths of the respondents included “Recruitment is not easy” (*n* = 32, 47.1%) and “Government values budget impact analysis more than cost-effectiveness analysis” (*n* = 31, 45.6%). In addition, among domestic respondents, “Difficulties in connecting theories and practices” and “HTA research is not valued” were also perceived as difficulties by more than two-fifths of the respondents. The differences in proportions of respondents who reported having difficulties with each assessed item among academia, government, and industry are presented in Table 3. In addition to those commonly reported difficulties, around one third of the respondents working for the government or for an industry also perceived “The views among industry, academia, and government are significantly different” as a difficulty they faced.

**Table 3:** Difficulties encountered by respondents in conducting HTA

Difficulties encountered	All respondents (*n* = 68)	Domestic respondents (*n* = 40)	Overseas respondents (*n* = 28)	Academia (*n* = 30)	Government (*n* = 23)	Industry (*n* = 15)
Lack of local data	36 (52.9%)	21 (52.5%)	15 (53.6%)	15 (50.0%)	18 (78.3%)	3 (20.0%)
Recruitment is not easy	32 (47.1%)	17 (42.5%)	15 (53.6%)	16 (53.3%)	11 (47.8%)	5 (33.3%)
Government values budget impact analysis more than cost-effectiveness analysis	31 (45.6%)	22 (55.0%)	9 (32.1%)	11 (36.7%)	9 (39.1%)	11 (73.3%)
Difficulties in connecting theories with practices	23 (33.8%)	18 (45.0%)	5 (17.9%)	10 (33.3%)	7 (30.4%)	6 (40.0%)
HTA research is not valued	20 (29.4%)	16 (40.0%)	4 (14.3%)	8 (26.7%)	6 (26.1%)	6 (40.0%)
The views among industry, academia and government are significantly different	20 (29.4%)	13 (32.5%)	7 (25.0%)	7 (23.3%)	8 (34.8%)	5 (33.3%)
Technical difficulties at the implementation level	17 (25.0%)	9 (22.5%)	8 (28.6%)	7 (23.3%)	6 (26.1%)	4 (26.7%)
Existing personnel transformation (e.g., transfer from other areas to HTA) is difficult	14 (20.6%)	8 (20.0%)	6 (21.4%)	5 (16.7%)	6 (26.1%)	3 (20.0%)
The cost of training new recruits is too high	12 (17.6%)	6 (15.0%)	6 (21.4%)	5 (16.7%)	6 (26.1%)	1 (6.7%)
Other	8 (11.8%)	2 (5.0%)	6 (21.4%)	3 (10.0%)	5 (21.7%)	0 (0.0%)

## Discussion

Health technology assessment has become more and more important in many countries over the past few years [8], and demand for HTA professionals has increased in academia, governments, and industries. Until now, there has been little research on the cultivation of HTA talent in Taiwan or in Asia. We hope that the findings of this study provide useful information for HTA trainers and educators.

One ultimate goal of training and education is to build up students’ competencies as required by relevant jobs. We found that across academia, government, and industry positions, pharmacoeconomics and alike courses were perceived to be the most important and most helpful among all the courses assessed. Indeed, analytical methodologies and skills such as cost-effectiveness and budget impact analyses are essential for conducting HTAs [9]. Even so, HTA is a multidisciplinary process that involves various kinds of analyses and considers multiple aspects of implications (including ethical, legal, and social) [10] in order to inform reimbursement or health policy decisions. As such, conducting an HTA or designing an HTA training program should also include the political and sociocultural views of health technology. Moreover, during the process of generating and using HTA evidence, future HTA workers will also require skills in management, coordination and communication [11].

The study findings and respondents’ written feedback also suggest our TMU HTA program can be improved as follows: (1) incorporate courses that provide students with the knowledge and skills needed to conduct assessments (systematic reviews, meta-analyses, and large database analyses) and then write professional HTA reports; (2) include other elective modules that may also improve HTA competency, such as Health Care System and Policy, Survival Analysis, Bayesian thinking, Pharmacoepidemiology, and Healthcare Financing; (3) provide opportunities to broaden and deepen students’ scope of HTA learning by incorporating experiences like internships at HTA-focused institutions.

As the importance and use of HTA are increasing, different organizations have attempted to discuss and delineate the competencies required by HTA professionals. For example, in 2014, the International Network of Agencies for Health Technology Assessment (INAHTA) convened a workshop on current experiences in capacity building in HTA. A few essential hard/scientific skills (e.g., search and critical appraisal of literature, statistics, economic analysis) and soft skills (e.g., management, writing, interaction with team members, consensus building) were identified by the workshop participants [12]. As a follow-up, Mueller and colleagues conducted four interlinked research activities (including handbook/toolkit reviews, workshops, and surveys via questionnaires) between 2016 and 2019 and identified a list of competencies needed for HTA [13]. Currently, HTA programs in educational institutions around the world are diversified, and the consensus on core competencies is still being established among the stakeholders involved in HTA. We hope our experience with the TMU HTA program and the findings of the present study contribute to the existing literature and also provide useful information for future HTA talent cultivation.

Given resource constraints, economic evaluations on whether to adopt a new health technology or discontinue an old one in the health care system have become an integral component of the resource allocation decision making process. Budget impact analysis (BIA) and cost-effectiveness analysis (CEA) are thus increasingly required by the authorities of health care sectors before reimbursement or formulary approval. Currently, in Taiwan, when applying for reimbursement by the national health insurance, manufacturers are required to provide budget impact analyses on new drugs and medical devices, but pharmacoeconomic evaluations are optional. However, in order to advance HTA in Taiwan, manufacturers get incentivized to conduct local pharmacoeconomic analyses to get up to a 10% price mark-up. While BIA assesses affordability, CEA estimates value for money. The resources needed to conduct a CEA and its level of complexity are certainly no less than, and are most likely greater than, those for a BIA. Nevertheless, as our results indicate, HTA experts, particularly those in the industrial sector, believe the government values BIA more than CEA, which is a difficulty, and probably a disappointment, they are facing.

Our survey found that a lack of local data is a major challenge for HTA, and this has also been observed in other countries, such as India and those in Latin America. Rosselli et al. reported that the limited availability of local data was a hurdle for conducting HTA in Latin America [14]. Indeed, most of their survey respondents acknowledged the crucial role of local data in HTA implementation and were willing to invest in local databases. Moreover, Downey et al. found that, in India, there was a significant lack of certain local data needed to conduct HTA, particularly data relating to cost, service use, and quality of life [15]. As HTA is country-specific, HTA proponents will have to overcome gaps in local data in each individual country. Governments should make a greater effort to encourage and assist academic and industrial institutions to generate local cost, epidemiological, and health utility data. Furthermore, both the standardization and transparency of data collection mechanisms need to be established.

There are several limitations to this study. First, although reminder emails were sent twice, the response rate was unsatisfactory. Second, despite our efforts to search for experts in HTA-related fields and the ample experience of those who responded (an average of 11.3 years of relevant working experience), the study findings may not have represented the opinions of all HTA experts.

## Conclusion

Given the increasing demand for HTA proficiency, we hope our study findings will provide a better understanding of the essential components of HTA talent cultivation and will serve as a reference for developing and modifying HTA education and training programs.

## Supplementary Information

**Additional file1.** Survey on the Health Technology Assessment (HTA) Credit Program and the Core Competencies of HTA Proficiency.

## Data Availability

The datasets used and/or analyzed during the current study are available from the corresponding author on reasonable request.
