# Supplementary material for: Talent cultivation in health technology assessment: an expert survey
Source: BMC Med Educ. 2022 Mar 8;22:157. doi: 10.1186/s12909-022-03214-z (PMC8903637; doi:10.1186/s12909-022-03214-z)
Supplement: Supplementary file 1 — Additional file1. Survey on the Health Technology Assessment (HTA) Credit Program and the Core Competencies of HTA Proficiency. [file 12909_2022_3214_MOESM1_ESM.docx]

**Taipei Medical University**

Survey on the Health Technology Assessment (HTA) Credit Program and the Core Competencies of HTA Proficiency

Health technology assessment (HTA) refers to the systematic evaluation of the properties, effects, and impacts of health technology (including medicines, medical devices, and procedures) by methods such as Comparative Effectiveness Analysis, Cost-Effectiveness Analysis, and Budget Impact Analysis. The assessment results can assist the policy/decision maker in identifying cost-effective health technologies and avoiding the use of controversial items with the aim of effectively using medical resources.

HTA is still in an emerging stage in Taiwan and at present is mainly used to assess whether new drugs should be included in the National Health Insurance’s reimbursement coverage. In view of the increasing demand for HTA proficiency, with the sponsorship and guidance of the Center for Drug Evaluation (CDE, Taiwan), we integrated relevant courses and established the HTA credit program (hereafter referred to as the HTA program) at Taipei Medical University in 2016 for our graduate and undergraduate students, with an aim of promoting domestic HTA research and cultivating talent in this field.

In order to better understand the requirements for HTA proficiency, this questionnaire invites experts and supervisors in HTA fields to make suggestions. The survey findings will serve as a reference for the modification of the HTA program in order to further HTA proficiency. The results of this survey are for academic education only. We gratefully appreciate your participation. Please do not hesitate to contact us if you have any questions:

Research Center for Pharmacoeconomics, College of Pharmacy, Taipei Medical University

Phone: (+8862) 27361661 ext.6190

E-mail：tmurcp@gmail.com

Best regards,

Yu Ko, PhD

Associate Professor

Taipei Medical University

**Part One**

| **I. Assessment of the importance of each course for HTA proficiency** | | | | | | | |
| --- | --- | --- | --- | --- | --- | --- | --- |
| The following are the courses listed in the HTA program. Click on the name of each subject to see the actual content of the course. Please assess the importance of each course for the development of HTA proficiency. | | | | | | | |
| **No.** | **Course name** | **Highly important**  **(5)** | **Important**  **(4)** | **Average**  **(3)** | **Not important**  **(2)** | **Highly not important**  **(1)** | **Not sure** |
| Comments (please suggest courses that need to be added to the HTA program): | | | | | | | |

| **II. Assessment of the usefulness of each course for HTA proficiency in various fields** | | | | | | | | | | | | | | | | | | | |
| --- | --- | --- | --- | --- | --- | --- | --- | --- | --- | --- | --- | --- | --- | --- | --- | --- | --- | --- | --- |
| 1 | Please tick the area that best describes your field, and select five courses from the list below that are most helpful to HTA personnel in your field. (1~5, 1 is the most helpful). | | | | | | | | | | | | | | | | | | |
| **Industry** | | Health economics and outcomes research department (pharmaceutical company)_ |  |  |  |  |  |  |  |  |  |  |  |  |  |  |  |  |  |
|  |  | Market access and pricing department (pharmaceutical company) |  |  |  |  |  |  |  |  |  |  |  |  |  |  |  |  |  |
|  |  | Health economics / market analysis  (consultant company) |  |  |  |  |  |  |  |  |  |  |  |  |  |  |  |  |  |
| **Government/Independent Assessment Organization** | | Government |  |  |  |  |  |  |  |  |  |  |  |  |  |  |  |  |  |
|  |  | Independent assessment organization, such as NICE (UK), CADTH (Canada), and PBAC (Australia) |  |  |  |  |  |  |  |  |  |  |  |  |  |  |  |  |  |
|  |  |  |  |  |  |  |  |  |  |  |  |  |  |  |  |  |  |  |  |
| **Academic/ Research Units** | | Professor / associate professor / assistant professor |  |  |  |  |  |  |  |  |  |  |  |  |  |  |  |  |  |
|  |  | Research Fellow / Associate Research Fellow / Assistant Research Fellow |  |  |  |  |  |  |  |  |  |  |  |  |  |  |  |  |  |

| 2. Please assess the extent to which the following activities are helpful for the development of HTA proficiency. | | | | | | | |
| --- | --- | --- | --- | --- | --- | --- | --- |
| **No.** | **Item** | **Very Helpful**  **(5)** | **Helpful**  **(4)** | **Average**  **(3)** | **Not Very Helpful**  **(2)** | **Not At All Helpful**  **(1)** | **Not Sure** |
| 1 | Invite domestic and overseas professionals to offer short-term training courses (e.g., one-day workshops) on HTA-related topics | **□** | **□** | **□** | **□** | **□** | **□** |
| 2 | Invite HTA-related academic/industry/public sector experts to give a talk | **□** | **□** | **□** | **□** | **□** | **□** |
| 3 | Visit HTA-related agencies/organizations | **□** | **□** | **□** | **□** | **□** | **□** |
| 4 | Internship at an institution related to HTA | **□** | **□** | **□** | **□** | **□** | **□** |
| 5 | Establish HTA student clubs/associations | **□** | **□** | **□** | **□** | **□** | **□** |

| **Future Needs for HTA Talent** | | | | | | | |
| --- | --- | --- | --- | --- | --- | --- | --- |
| **No.** | **Item** | **Significantly Increase**  **(5)** | **Slightly Increase**  **(4)** | **No Change**  **(3)** | **Slightly Decrease**  **(2)** | **Significantly Decrease**  **(1)** | **Not Sure** |
| 1 | How do you think the demand for HTA proficiency in **pharmaceutical companies** will change in your country? | **□** | **□** | **□** | **□** | **□** | **□** |
| 2 | How do you think the demand for HTA proficiency in **consulting companies** will change in your country? | **□** | **□** | **□** | **□** | **□** | **□** |
| 3 | How do you think the demand for HTA proficiency in **the government/independent assessment organizations** will change in your country? | **□** | **□** | **□** | **□** | **□** | **□** |
| 4 | How do you think the demand for HTA proficiency in **academic/research units** will change in your country? | **□** | **□** | **□** | **□** | **□** | **□** |
| **Please provide your comments on HTA demands and talent cultivation (e.g., Are the supply and demand for HTA personnel balanced? What advice would you give regarding the cultivation of HTA talent?)** | | | | | | | |

| **What are the difficulties faced in YOUR HTA field? (please select all that apply)** | | |
| --- | --- | --- |
| 1 | Existing personnel transformation (e.g., transfer from other areas to HTA) is difficult | **□** |
| 2 | Recruitment is not easy | **□** |
| 3 | The cost of training new recruits is too high | **□** |
| 4 | Difficulties in connecting theories with practices | **□** |
| 5 | The views among industry, academia and government are significantly different | **□** |
| 6 | Technical difficulties at the implementation level | **□** |
| 7 | HTA research is not valued | **□** |
| 8 | Government values budget impact analysis more than cost-effectiveness analysis | **□** |
| 9 | Lack of local data | **□** |
| 10 | Other (please specify: ____) | |

**Part two: Personal Information**

1. What is the name of the company/organization you currently work for?
2. What is the name of the department in which you currently serve? ______________________
3. How long have you worked in HTA-related fields (including all past years in HTA, not limited to the same company)? __________years
4. Gender: □ male □ female
5. Age: ___________ years old
6. What is your highest level of education: □ junior high school and below □ high school / vocational school □ university (including colleges) □ masters and equivalent □ PhD and equivalent
7. Which country do you live in now? ______________________

**Part three: Other suggestions: ________________________________**

**～Thank you for completing the questionnaire～**
